# Widely targeted metabolomics reveals stamen petaloid tissue of *Paeonia lactiflora* Pall. being a potential pharmacological resource

**DOI:** 10.1371/journal.pone.0274013

**Published:** 2022-09-02

**Authors:** Xianghui Liu, Ye Chen, Jingxiao Zhang, Yifan He, Huiyuan Ya, Kai Gao, Huizhi Yang, Wanyue Xie, Lingmei Li

**Affiliations:** 1 School of Food and Drug, Henan Functional Cosmetics Engineering Technology Research Center, Luoyang Normal University, Luoyang, Henan, China; 2 Institute of Regulatory Science, Beijing Technology and Business University, Beijing, China; 3 Peony Institute, Luoyang Academy of Agriculture and Forestry Sciences, Luoyang, Henan, China; Lahore College for Women University, PAKISTAN

## Abstract

*Paeonia lactiflflora* Pall. has a long edible and medicinal history because of the very high content of biologically active compounds. However, little information is available about the metabolic basis of pharmacological values of *P*. *lactiflora* flowers. In this study, we investigated metabolites in the different parts of *P*. *lactiflora* flowers, including petal, stamen petaloid tissue and stamen, by widely targeted metabolomics approach. A total of 1102 metabolites were identified, among which 313 and 410 metabolites showed differential accumulation in comparison groups of petal vs. stamen petaloid tissue and stamen vs. stamen petaloid tissue. Differential accumulated metabolites analysis and KEGG pathway analysis showed that the flavonoids were the most critical differential metabolites. Furthermore, difference accumulation of flavonoids, phenolic acids, tannins and alkaloids might lead to the differences in antioxidant activities and tyrosinase inhibition effects. Indeed, stamen petaloid tissue displayed better antioxidant and anti-melanin production activities than petal and stamen through experimental verification. These results not only expand our understanding of metabolites in *P*. *lactiflora* flowers, but also reveal that the stamen petaloid tissues of *P*. *lactiflora* hold the great potential as promising ingredients for pharmaceuticals, functional foods and skincare products.

## Introduction

*Paeonia lactiflora* Pall., a species with perennial herbaceous flower, has a long history of cultivation in botanical gardens. This traditional famous flower of China is renowned as the king of flowers together with tree peony, having great ornamental value. In addition, *P*. *lactiflora* is also an excellent plant resource that has the concomitant function of both medicine and foodstuff in China. The dried roots of *P*. *lactiflora*, also known as Paeoniae Radix Alba (PA) and Radix Paeoniae Rubra (PR), have been used for centuries as traditional Chinese medicines [1] in the treatment of various diseases [2–4]. *P*. *lactiflora* seed oil, which is rich in unsaturated fatty acids and γ-tocopherol, shows good healthcare function [5].

In recent years, there is growing evidence that *P*. *lactiflora* flowers not only possess ornamental and edible application [6], but also present high nutritional value and healthcare function with antioxidant [7,8], anti-inflammatory [9,10] and anti-bacterial [11] properties. Modern biology studies have revealed that *P*. *lactiflora* flowers had positive effects on modulating female endocrine system [12,13]. Moreover, the total glucosides of *P*. *lactiflora* flowers can remarkably reduce the serum uric acid levels in a mouse model of hyperuricaemi by inhibiting the synthesis of uric acid in kidney [14]. Furthermore, *P*. *lactiflora* flowers extract can inhibit the development of bladder cancer via inducing apoptosis and cell cycle arrest [15].

A growing number of studies have confirmed that many pharmacological effects, which are not limited to the described above, are closely correlated with paeoniflorin, benzoylpaeoniflorin, galloylpaeoniflorin and their derivatives in *P*. *lactiflora* [16,17]. Because of the distinct biosynthetic abilities of terpenoid and paeoniflorin biosynthesis [18], those compounds are always the hot topics of active ingredients in *P*. *lactiflora* flowers. In addition, flavonoids [19–22], phenolic acids and tannins [23], are often detected and analyzed through high-performance liquid chromatography (HPLC), LC/GC-MS and Nuclear Magnetic Resonance (NMR). Previous analytical techniques are mostly based on standards, with high data accuracy and reliability, but limited coverage of metabolites. Current researches mostly focus on active compounds in different cultivars [22,24] and different blooming stages [20] of *P*. *lactiflora* flowers, while very little attention has been paid to the biologically active substances in different parts of the flowers.

To date, there are more than 600 species of cultivars all over the worldwide, which can be subdivided into two categories, namely simple-petal and double-petals [25]. Indeed, double-petals can be attributed to the formation of stamen petaloid [26], which may be regulated by a complicated genetic pathway [27–29]. This phenomenon may lead to a highly complex period of biochemical changes from stamen transform to petaloid tissue. Transcriptome sequencing reveals coordinated expression of anthocyanin biosynthetic genes mediating stamen petaloid tissue formation and color change in stamen petalody process of *P*. *lactiflora* [30,31]. In addition, Danlong Jing et al. [32] have found that the concentrations of endogenous hormones, including indoleacetic acid, kinetin and GA3, showed significant differences between petaloid tissue and petal. However, there has been limited information about functional metabolites change during this process.

Antioxidant activity, a major function of *P*. *lactiflora* flowers, is closely related to the prevention and treatment of age-related diseases, cancer and diabetes mellitus [33,34]. In recent years, as the most important functional demand in the market, antioxidant activity always has been the research hotspot in food [35,36], drug [37,38], health product and cosmetic industries [39]. Widely targeted metabolome, mainly based on ultrahigh performance liquid chromatography coupled to triple quadrupole mass spectrometry (UPLC-QQQ-MS) techniques, has become a powerful approach combining with the advantages of targeted metabolomics [40]. Recently, this method has been widely applied in correlation analysis between plant metabolites and antioxidant activity [41–43].

In this research, we analyzed metabolites profiles and detected compositions variations in the petal, stamen petaloid tissue and stamen of *P*. *lactiflora* flowers through widely targeted metabolome method. Meanwhile, a comparative study was conducted to analyze the functional components associated with antioxidant activities and verify their antioxidant capacity and tyrosinase inhibition activity. This study can provide a theoretical reference for the future application of *P*. *lactiflora* flowers, particularly the stamen petaloid tissue, as nutraceuticals or functional cosmetics.

## Material and methods

### Plant materials

**Fig 1:**
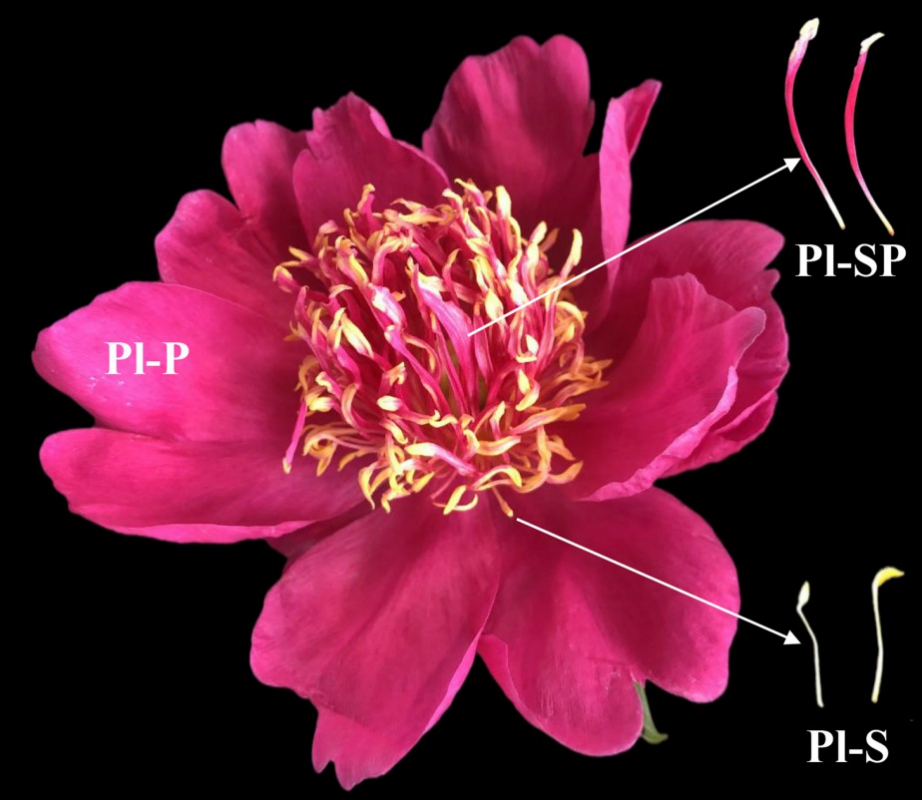
Flower morphology of *P*. *lactiflora*.

A double-petals cultivar of *P*. *lactiflora*, ‘Zijinlian’, was used in this study (Fig 1). All the samples were collected from peony germplasm resource of Luoyang Academy of Agriculture and Forestry Sciences, Henan, China (112°48’N, 34°63’E) in May 2021. The samples were directly frozen in liquid nitrogen and stored at -80°C until metabolites extraction. Petal, stamen petaloid tissue and stamen of *P*. *lactiflora* flowers were named by Pl-P, Pl-SP and Pl-S respectively. For each sample, three biological replicates were independently analyzed.

### Sample preparation

Biological samples were freeze-dried by vacuum freeze-dryer (Scientz-100F). The freeze-dried samples were crushed using a mixer mill (MM 400, Retsch) with a zirconia bead for 1.5 min at 30 Hz. One-hundred milligrams of lyophilized powder was dissolved with 1.2ml 70% aqueous methanol, vortexed 30 seconds every 30 minutes for 6 times in total, then placed in a refrigerator at 4°C overnight. Following centrifugation at 12000rpm for 10 min, the extracts were filtrated (SCAA-104, 0.22μm pore size; ANPEL, Shanghai, China, http://www.anpel.com.cn/) before Ultra Performance Liquid Chromatography Tandem Mass Spectrometry (UPLC-MS/MS, UPLC, SHIMADZU Nexera X2, https://www.shimadzu.com.cn/; MS/MS, Applied Biosystems 4500 QTRAP, http://www.appliedbiosystems.com.cn/) analysis.

### UPLC conditions and ESI-QTRAP-MS/MS conditions

The sample extracts were analyzed using an UPLC-ESI-MS/MS system. The analytical conditions were as follows, UPLC: column, Agilent SB-C18 (1.8 μm, 2.1 mm × 100 mm); the mobile phase consisted of solvent A, pure water with 0.1% formic acid, and solvent B, acetonitrile with 0.1% formic acid. Sample measurements were performed with a gradient program that employed the starting conditions of 95% A and 5% B. Within 9 min, a linear gradient to 5% A, 95% B was programmed, and a composition of 5% A and 95% B was kept for 1 min. Subsequently, a composition of 95% A and 5.0% B was adjusted within 1.10 min and kept for 2.9 min. The column oven was set to 40°C. The injection volume was 4 μL. The effluent was alternatively connected to an electrospray ionization (ESI)-triple quadrupole-linear ion trap (QTRAP)-MS.

Linear ion trap (LIT) and triple quadrupole (QQQ) scans were acquired on a triple quadrupole-linear ion trap (Q-TRAP) mass spectrometer, AB4500 Q-TRAP-UPLC/MS/MS System, equipped with an ESI Turbo Ion-Spray interface, operated in positive and negative ion mode and controlled by Analyst 1.6.3 software (AB Sciex, Concord, ON, Canada). The ESI source operation parameters were as follows: ion source, turbo spray; source temperature 550°C; ion spray voltage 5500 V (positive ion mode)/-4500 V (negative ion mode); ion source gas I, gas 28 II and curtain gas were set at 50, 60, and 25.0 psi, respectively; the collision gas was high. Instrument tuning and mass calibration were performed with 10 and 100 μmol/L polypropylene glycol solutions in QQQ and LIT modes, respectively. QQQ scans were acquired as multiple reaction monitoring (MRM) experiments with collision gas (nitrogen) set to medium. Declustering potential (DP) and collision energy (CE) for individual MRM transitions were done with further DP and CE optimization. A specific set of MRM transitions was monitored for each period according to the metabolites eluted within this period.

### Qualitative and quantitative analysis of metabolites

Based on the self-built database MWDB V2.0 (Metware Biotechnology Co., Ltd. Wuhan, China) and public databases, such as MassBank (http://www.massbank.jp), KNApSAcK (http://kanaya.naist.jp/KNApSAcK), HMDB (Human Metabolome Database, http://www.hmdb.ca), and METLIN (Metabolite Link, http://metlin.scripps.edu/index.php), metabolite information of samples was matched with subjected existing mass spectrometry databases to qualitative analysis. The matching parameters, including Q1 precise molecular mass, secondary fragmentation, retention time and isotope peak were used in the intelligent matching method explored by Metware. In addition, MS1 tolerance and MS2 tolerance were set to 20 ppm and 20ppm to ensure that the metabolites could be identified accurately.

Metabolite quantification was performed by MRM of triple quadrupole mass spectrometry. In the MRM mode, the quadrupole filtered the precursor ions of the target substance and excluded the ions corresponding to other molecular weights to eliminate interference. After obtaining the metabolite mass spectrometry data, peak area integration was performed using Multi Quant (version 3.0.2, AB SCIEX, Concord, ON, Canada). Chromatographic peak area was used to determine the relative metabolite contents. The original abundance of metabolites was log-transformed to normalize the data and for homogeneity of variance.

### Metabolome data processing and analysis

Principal component analysis (PCA), and hierarchical cluster analysis (HCA) were performed to the unit variance scaling data using R software (http://www.r-project.org/). The orthogonal partial least squares-discriminant analysis (OPLS-DA) was performed using MetaboAnalystR package of R software. And the modeling was validated through a permutation analysis with the model parameters (Q2 and R2Y) both close to 1. Variable importance in projection (VIP) values of all metabolites from the OPLS-DA were extracted using the first component. The metabolites satisfying the following two criteria were selected as differential metabolites of the comparison groups (Pl-P vs Pl-SP, Pl-S vs Pl-P and Pl-S vs Pl-SP): (i) fold change ≥ 2 and fold change ≤ 0.5; (ii) VIP ≥ 1. The screening of different metabolites was visualized in the form of the volcano plot. The Venn diagram was built to show the relationship between different metabolites in each comparison group.

Differential metabolites were annotated and classified using the Kyoto Encyclopedia of Genes and Genomes (KEGG) Pathway database (http://www.kegg.jp/kegg/pathway.html). Pathways with significantly regulated metabolites were compared to the background and defined by both a hypergeometric test and a threshold of p-value < 0.05.

### Antioxidant capacity analysis and tyrosinase inhibition assay

The fresh samples of Pl-P, Pl-SP and Pl-S were separated and crushed, respectively. One hundred micrograms of samples were ultrasonically extracted with 1 mL ethanol at 45°C for 20 min. The samples were centrifuged at 5000 rpm for 10 min. The supernatant was passed through a 0.22 μm filter membrane. These extracts were used for antioxidant capacity analysis and tyrosinase inhibition assay with 3 parallel samples.

#### Antioxidant capacity analysis

In this study, ferric reducing antioxidant power (FRAP) assay and 2,2’-diphenyl-1-picrylhydra-zyl (DPPH) free radical scavenging test were used to compare the antioxidant effects of Pl-P, Pl-SP and Pl-S. All the experiments were kept under subdued light. The FRAP assay was conducted using a total antioxidant capacity assay kit (Micro total antioxidant capacity (T-AOC) assay kit, 100T/96S, Solarbio, Beijing, China). Briefly, 225 μL FRAP solution and 7.5 μL extract (or distilled water) were mixed in a 96-well microplate, then distilled water was added to a total of 255 μL. The mixture was mixed and kept for 10 min at room temperature under dim light, then the absorbance at 593 nm was measured by a microplate reader (PE Victor Nivo, PerkinElmer, Massachusetts, USA). ΔA was proportional to the concentration of Fe^2+^ (μmol/L). The results were expressed as micromole Fe^2+^ equivalents per gram fresh sample (μmol/g).

FRAP=34×x÷c (1)

where x was the concentration of Fe^2+^ corresponds to the ΔA of sample (μmol/L), c was the concentration of sample (g/L).

The DPPH scavenging activity was measured by a method described by Lars Müller et al. [44], with certain modifications. Briefly, 200 μL DPPH solution (or ethanol) and 50 μL extract were added to a 96-well microplate. After 30 min at room temperature under dim light, the absorbance was measured by the same instrument at 519 nm. The percentage of scavenging was calculated as follows:

$$\text{DPPH}\;\text{scavenging}\;\text{rate}=[1-({\text{D}}_{\text{x}}-{\text{D}}_{\text{x}0})÷{\text{D}}_{0}]\times 100\%$$ (2)

where D_0_ was the absorbance at 517nm with DPPH solution, D_x_ was the absorbance at 517 nm with the test sample and DPPH solution, D_x0_ was the absorbance at 517 nm with the test sample.

#### Tyrosinase inhibition assay

Tyrosinase plays a major role in melanin synthesis, and tyrosinase inhibition assay has been considered as a universal method to inhibit melanin production [45]. L-tyrosine and L-dopa were selected as substrate respectively to determine the inhibition of tyrosinase monophenolase and diphenolase activity with arbutin as a positive control. First, 50 μL of 1.5 mM L-tyrosine or L-dopa, 100 μL of phosphate buffer (PBS, pH 6.8), and 60 μL of PBS with or without the sample, were mixed. The mixture was preincubated at 37°C for 10 min before 40 μL of 250 units/mL mushroom tyrosinase was added, and the reaction was performed at 37°C for 25 min. Enzyme activity in different concentration of fresh sample was measured at 490 nm. The percentage of inhibition was calculated as follows:

$$\text{Inhibition}\;\text{ratio}=[1-(\text{T}-{\text{T}}_{0})÷(\text{C}-{\text{C}}_{0})]\times 100\%$$ (3)

where C was the absorbance at 490 nm with tyrosinase, but without the test sample; C_0_ was the absorbance at 490 nm without the test sample and tyrosinase; T was the absorbance at 490nm with the test sample and tyrosinase; and T_0_ was the absorbance at 490 nm with the test sample, but without tyrosinase.

According to the linear formula, we calculated the IC50 values of DPPH scavenging activity and tyrosinase inhibition effect. All the data was sorted and used to determine the significance differences of comparative groups Pl-P vs. Pl-SP and Pl-S vs. Pl-SP via T test (P<0.05).

## Results

### Metabolic profiling

**Fig 2:**
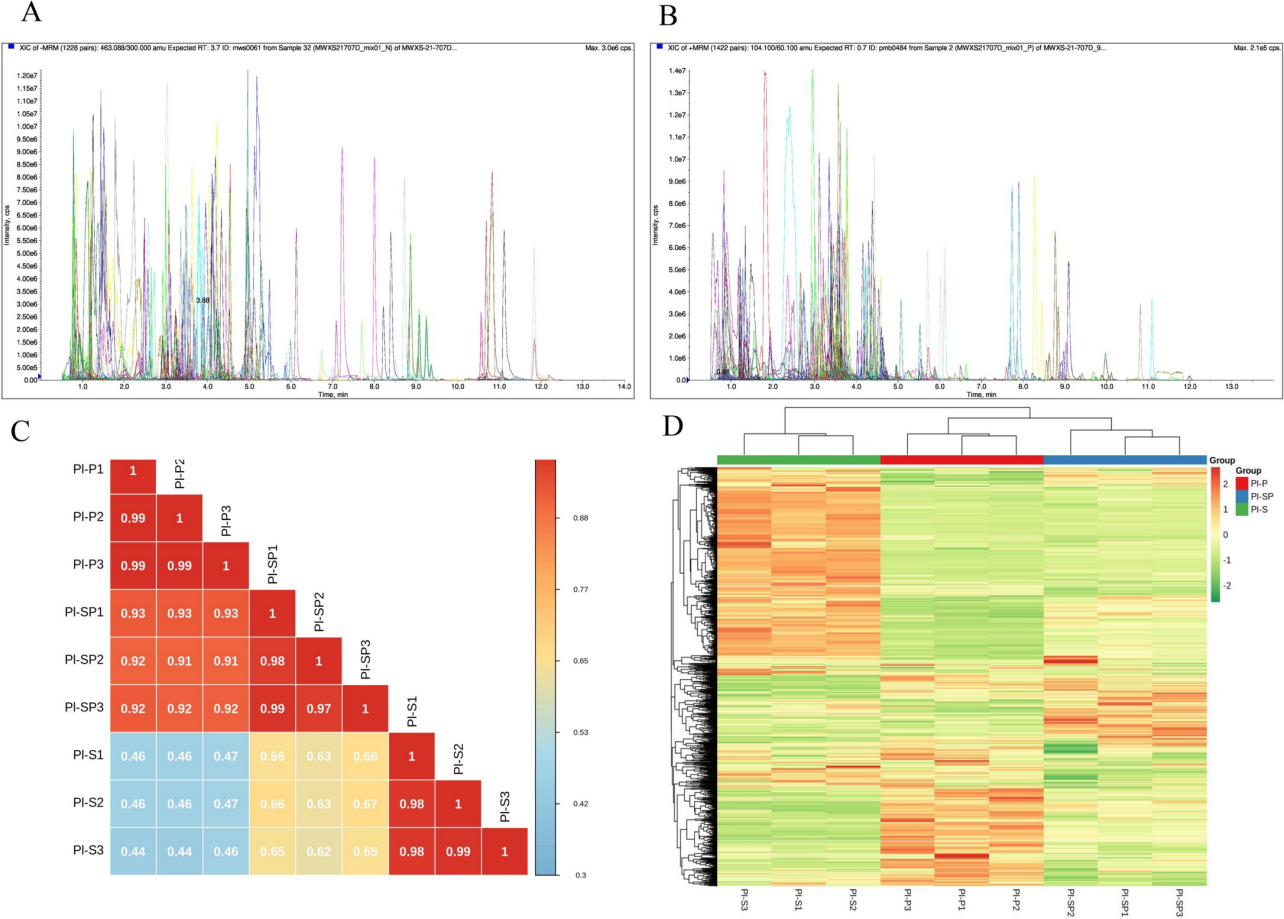
Qualitative and semi-quantitative analysis of metabolites. A-B. Multipeak mass spectral chromatogram of metabolites acquired in negative ion mode (A) and positive ion mode (B). C. Pearson’s correlation coefficients among all the samples. D. Cluster analysis of the identified metabolites.

To investigate the chemical composition of petal, stamen petaloid tissue and stamen of *P*. *lactiflora*, the metabolites were identified by UPLC-MS/MS analysis. The metabolites were quantitatively analyzed using software analyst under the multiple reaction monitoring modes (Fig 2A and 2B). Pearson’s Correlation Coefficient analysis showed that there were high interclass correlation coefficients (Fig 2C). Based on the hierarchical cluster analysis, 9 samples were clearly divided into three groups and the metabolites displayed different accumulation patterns between the intragroup samples (Fig 2D).

**Fig 3:**
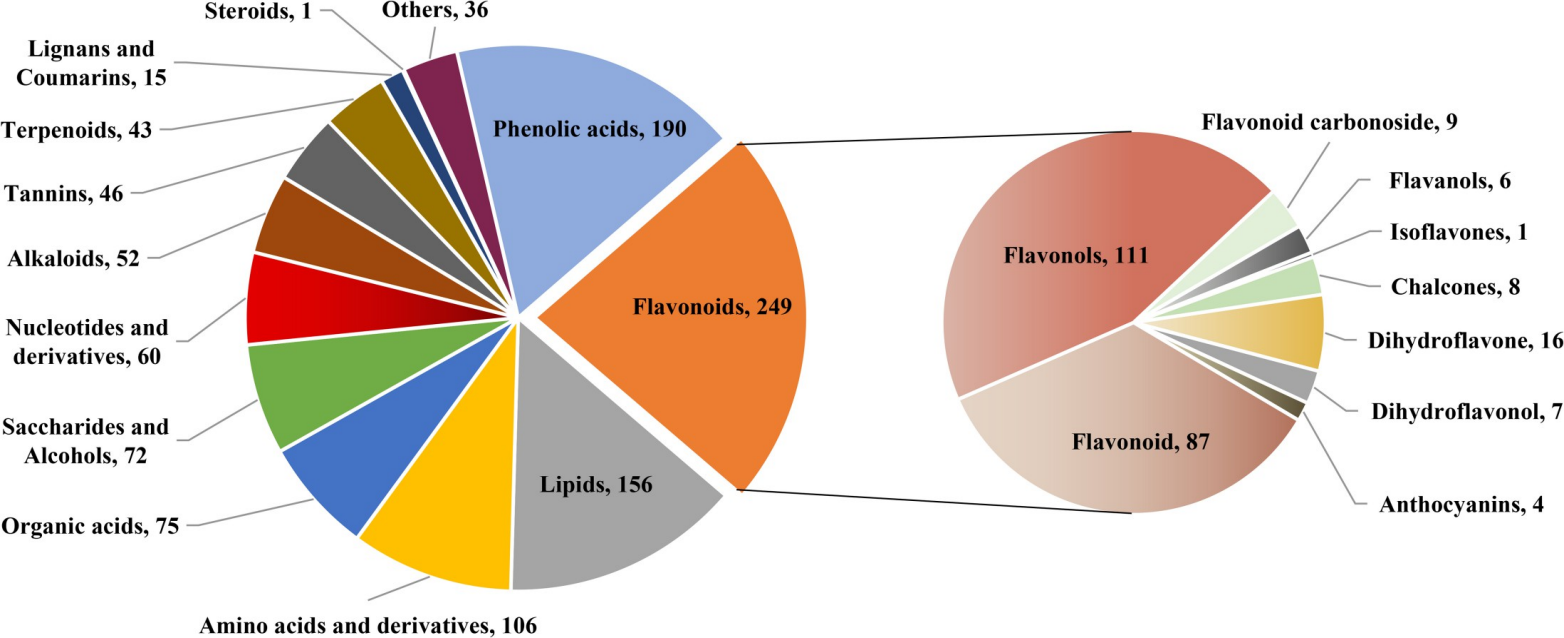
Classification of detected metabolites.

In total, 1102 metabolites were detected and could be categorized into more than ten different classes, including 249 flavonoids, 190 phenolic acids, 156 lipids, 106 amino acids and derivatives, 75 organic acids, 73 saccharides and alcohols, 60 nucleotides and derivatives, 52 alkaloids, 46 tannins, 43 terpenoids, 15 lignans and coumarins, one steroid, and 36 others. The flavonoids could be further categorized into nine classes, with flavonoid and flavonols occupying the majority (Fig 3). Detailed information of all identified metabolites was shown in S1 Table. Apart from common metabolites of monoterpenoids, such as paeoniflorin, oxypaeoniflorin, benzoylpaeoniflorin, lactiflorin, albiflorin and paeoniflorigenone, we also found abundant compounds of triterpene, triterpene saponin and terpene.

### PCA analysis

**Fig 4:**
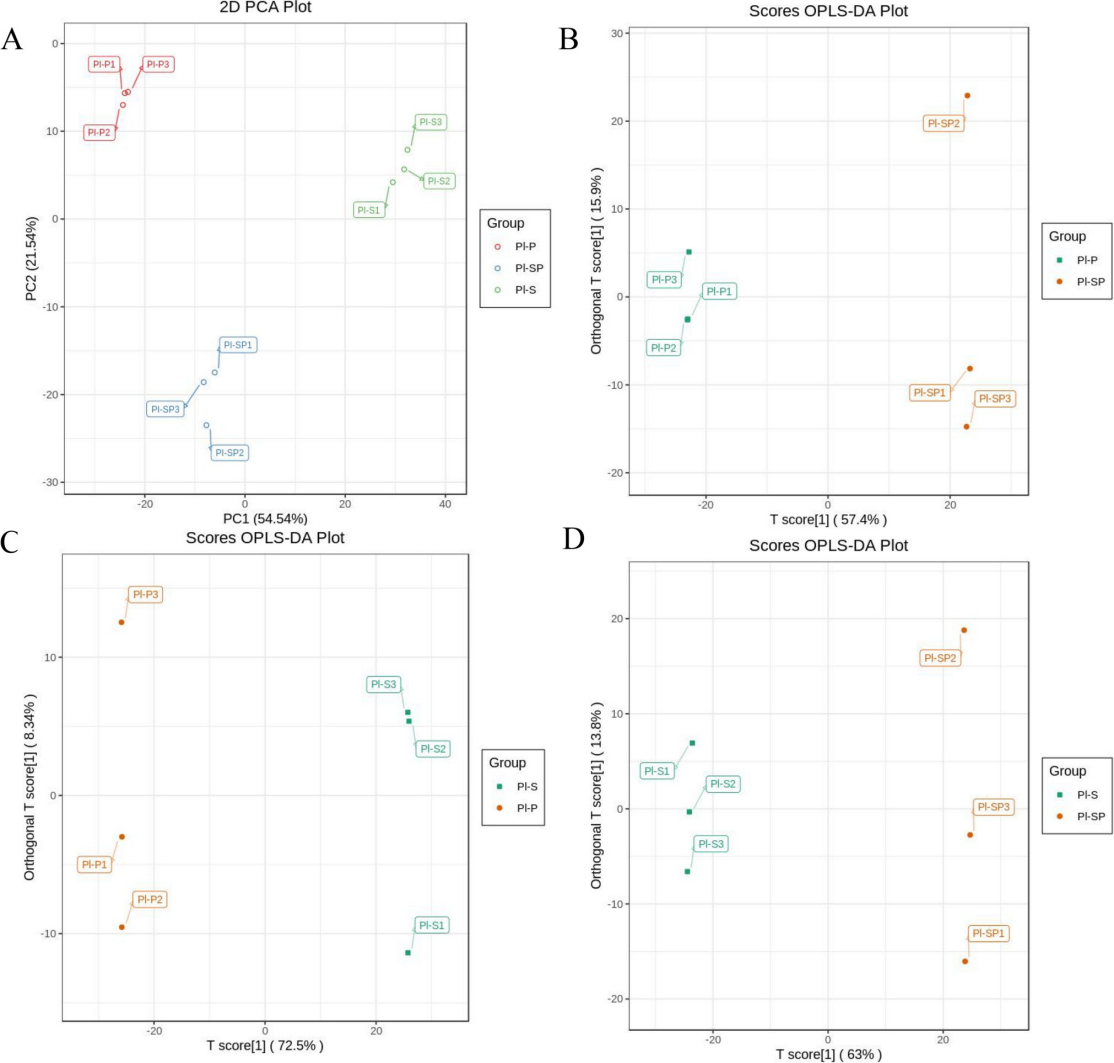
The identified metabolites analysis. A. PCA score plot. B-D. OPLS-DA model plots for the comparison groups Pl-P vs. Pl-SP (B), Pl-S vs. Pl-P (C), Pl-S vs. Pl-SP (D).

In order to further analyze the degree of variability in interclass samples and intragroup samples, the metabolites profile of nine samples was subjected to PCA score plot (Fig 4A). Two principal components model explained 77.08% (PC1 = 54.54%, PC2 = 21.54%) of the variance in total. The results showed that three groups were clearly separated, and three biological replicates of each group were compactly gathered together, indicating that the experiment were clustered well and clearly distinguished from other samples.

The OPLS-DA mode was used to screen the identified metabolites and evaluate the differential metabolites between intragroups (Fig 4B–4D). All the results exhibited an obvious separation between Pl-P and Pl-SP (Q2 = 0.974, R2X = 0.733, R2Y = 1), Pl-S and Pl-P (Q2 = 0.99, R2X = 0.808, R2Y = 1), Pl-S and Pl-SP (Q2 = 0.978, R2X = 0.767, R2Y = 1). The Q2 and R2Y values of all comparison groups exceeded 0.9, demonstrating that these models were stable and reliable and could be used to further identify the differential accumulated metabolites.

### Differential metabolites screening

**Fig 5:**
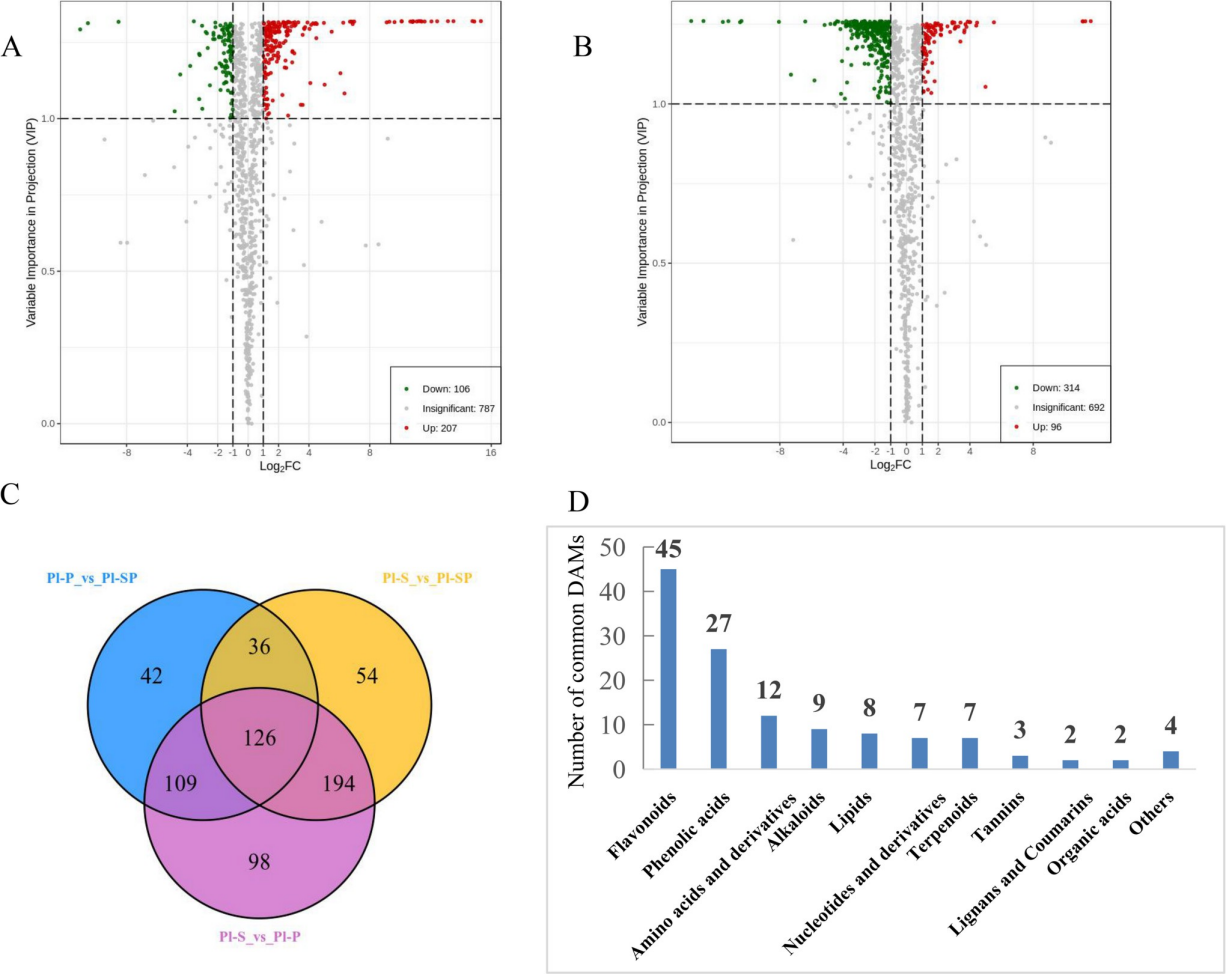
The differential metabolites analysis. A-B. Volcano plot of differential metabolites of the comparison groups Pl-P vs. Pl-SP (A), Pl-S vs. Pl-SP (B). C. The venn diagram of differential metabolites in three comparison groups. D. Number of different types of common differential metabolites.

To identify differential accumulated metabolites (DAMs) between comparison groups, a fold change ≥ 2 or ≤ 0.5 and VIP ≥ 1 were used as the screening criteria. The results showed that there were 313 DAMs between Pl-P and Pl-SP (207 up-regulated, 106 down-regulated) (Fig 5A and S2 Table), and 410 DAMs between Pl-S and Pl-SP (96 up-regulated, 314 down-regulated) (Fig 5B and S3 Table). The differential metabolites for the comparison groups of Pl-P vs. Pl-SP and Pl-S vs. Pl-SP were classified into 32 and 34 different categories (S4 Table). Significantly, the number of flavonoids and phenolic acids DAMs was well ahead of other categories. Besides, most flavonoids metabolites, including flavonols, chalcones, flavonoid, dihydroflavonol and dihydroflavone, were down-regulated in Pl-SP compared with Pl-S. Meanwhile, 126 common differential metabolites were identified in all the composition groups (Fig 5C) and were divided into 11 classes, including 45 flavonoids, 27 phenolic acids, 9 alkaloids, 8 lipids, 7 nucleotides and derivatives, 7 terpenoids, 3 tannins, 2 lignans and coumarins, and 4 others (Fig 5D).

### Putative antioxidant metabolites analysis

To further analyzed antioxidant components in Pl-S, Pl-P and Pl-SP, four classes of putative antioxidant metabolites, including flavonoids, phenolic acids, tannins and alkaloids, were observed from metabolome results (Table 1). Obviously, the up-regulated antioxidant DAMs vastly outnumbered down-regulated DAMs in Pl-P vs. Pl-SP, and the other composition groups Pl-S vs. Pl-SP showed the opposite. As shown in S1 Fig, flavones and flavonols were the major DAMs of flavonoids, and most of flavonoids showed higher relative concentrations in Pl-SP than Pl-P.

**Table 1:** Statistics of the DAMs with putative antioxidant in comparison groups.

Class	Pl-P vs. Pl-SP		Pl-S vs. Pl-SP	
	Up	Down	Up	Down
Flavonoids	52	39	23	77
Tannins	7	2	14	3
Phenolic acids	43	18	17	69
Alkaloids	16	2	2	15
**Sum**	118	61	56	164

**Fig 6:**
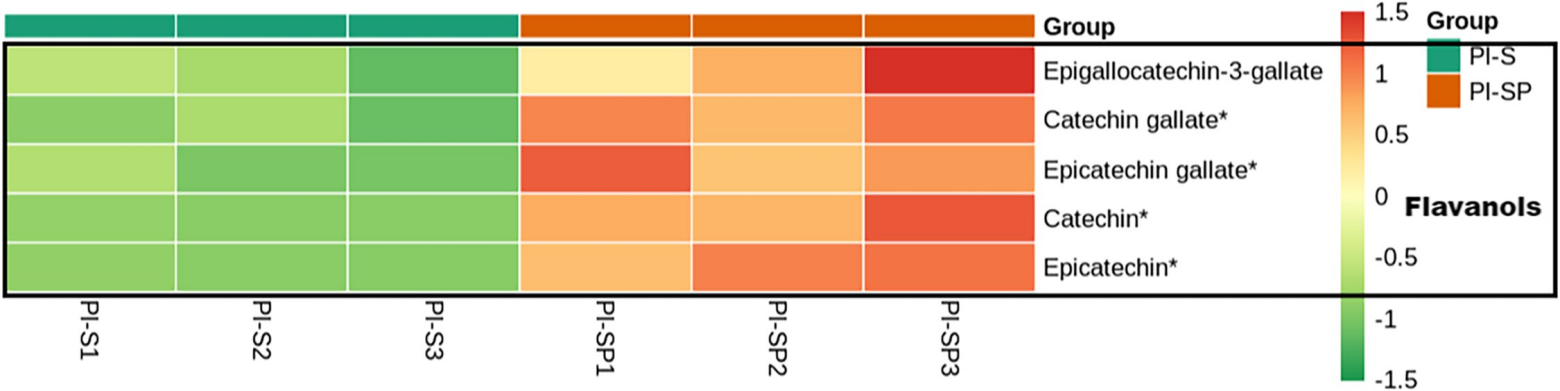
Heatmap of tea polyphenols in Pl-SP compare with Pl-S.

It was remarkable that catechin and epicatechin, catechin gallate, epicatechin gallate and epigallocatechin-3-gallate, as main components of tea polyphenols, had been detected in all the samples. Particularly, catechin and epicatechin were significantly up-regulated in Pl-SP compared with Pl-P and Pl-S (Fig 6).

Similarly, most of phenolic acids and alkaloids exhibited higher relative concentrations in Pl-SP than in Pl-P, and most tannins in Pl-SP were higher than Pl-P and Pl-S (S2 Fig). There were nine phenolic acids (including arbutin, 6’-p-coumarylarbutin, 4-O-(6’-O-glucosyl-4’’-hydroxybenzoyl)-4-hydro-xybenzyl alcohol, poliothrysoside, 2,6-dimethoxybenzaldehyde, 3-hydroxy-5-methylphenol-1-O-(6’-digalloyl) glucoside fertaric acid, and methyl anisate) were significantly accumulated in Pl-SP compared with Pl-P (Fold Change value>10^3^).

**Fig 7:**
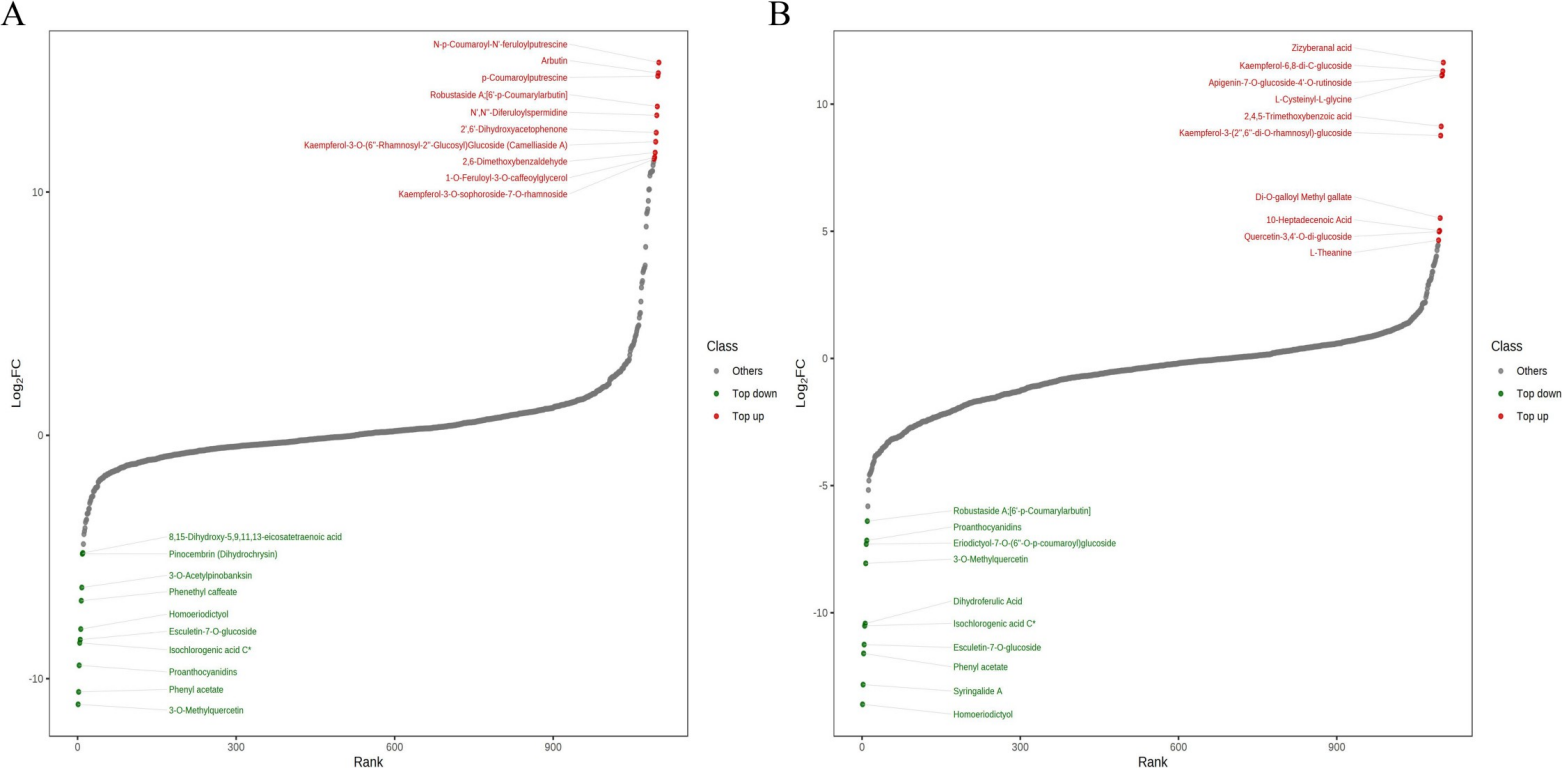
Dynamic distribution diagram of differences in metabolite content. A. Pl-P vs. Pl-SP. B. Pl-S vs. Pl-SP.

In order to show the overall metabolic differences more clearly and intuitively, the fold change values of the metabolites in the comparison groups were calculated, and arranged in an ascending sort order, following drawing a dynamic distribution diagram of the difference in metabolite content and the top 10 metabolites up-regulated and down-regulated were highlighted in Fig 7. The top 10 metabolites which were significantly up-regulated in Pl-SP (compared with Pl-P) included 4 phenolic acids, 3 alkaloids, 2 flavonoids and 1 lipid (Fig 7A). The relative concentration of these compounds showed higher enrichment degree in Pl-SP than in Pl-P (Fold Change value>10^3^). These metabolites could be considered as the representative differential metabolites of Pl-P vs. Pl-SP. The top 10 metabolites which were significantly up-regulated in Pl-SP (compared with Pl-S) were consisted of 5 flavonoids, 3 tannins, 1 terpenoid and 1 amino acids derivative (Fig 7B).

### Enrichment analysis and KEGG pathway

**Fig 8:**
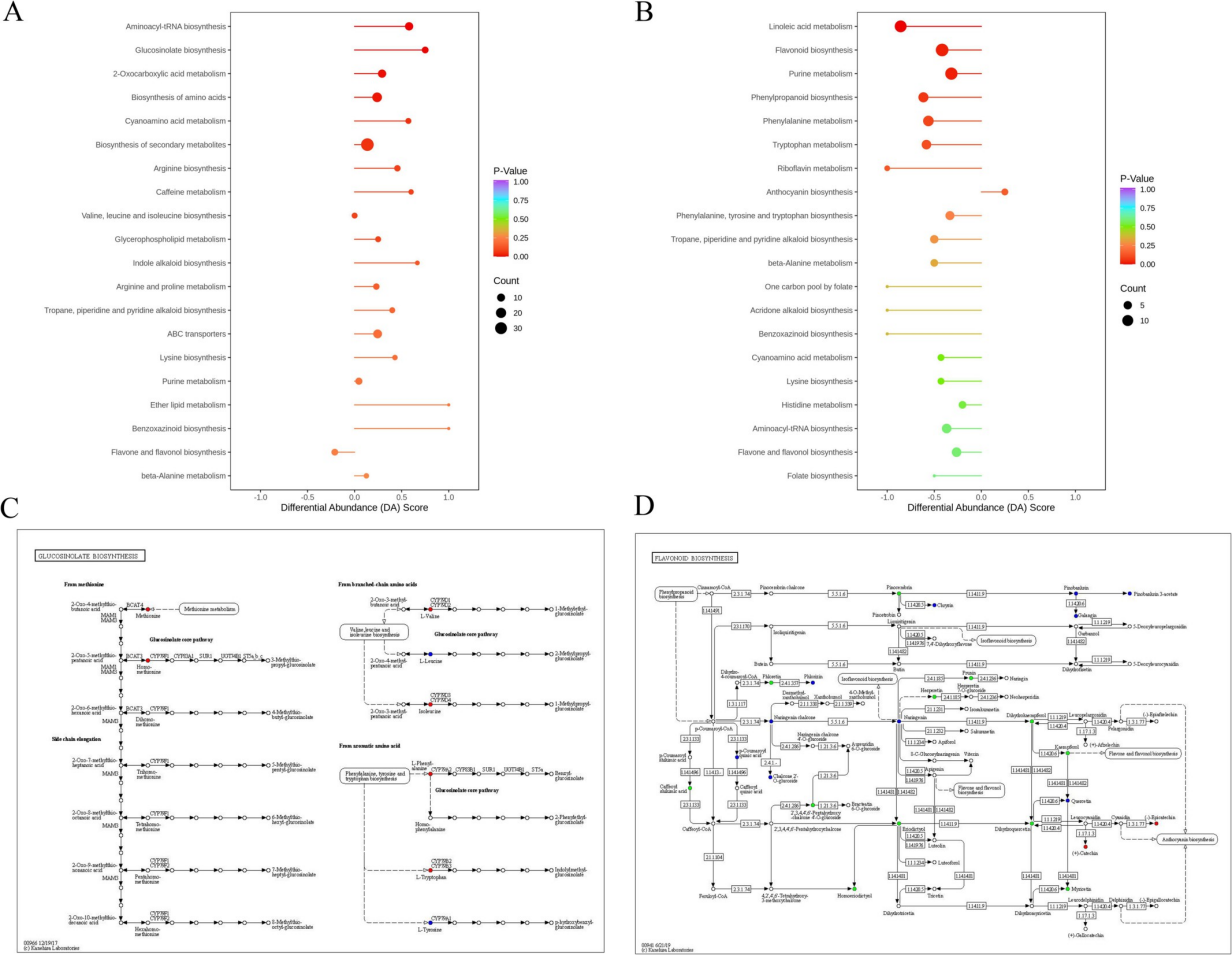
KEGG enrichment pathway differential metabolites. A-B. Metabolic enrichment pathway analysis in comparative groups of Pl-P vs. Pl-SP (A), and Pl-S vs. Pl-SP (B). C-D. KEGG pathway maps of glucosinolate biosynthesis (C) and flavonoid biosynthesis (D).

To identify the major pathways of DAMs in comparison groups of Pl-P vs. Pl-SP and Pl-S vs. Pl-SP, KEGG enrichment analysis was conducted in this study, and the enrichment results and detailed metabolic pathways were shown in Fig 8. The top enriched and significantly regulated KEGG pathways were mainly involved in aminoacyl-tRNA biosynthesis, glucosinolate biosynthesis, biosynthesis of amino acids and 2-Oxocarboxylic acid metabolism in the comparisons group of Pl-P vs. Pl-SP (Fig 8A). Whereas, in the comparison group of Pl-S vs. Pl-SP, the metabolic pathways of the differential metabolites mainly contained flavonoid biosynthesis, purine metabolism, linoleic acid metabolism (Fig 8B). Furthermore, amino acids were involved in three pathways, and played a crucial role in transforming stamen petaloid tissue to petal (Fig 8C). Fourteen DAMs, including catechin and epicatechin, were reflected in flavonoid biosynthesis pathway (Fig 8D).

### Antioxidant capacity analysis and tyrosinase inhibition assay

Based on the metabolomic analysis results, most antioxidant metabolites in Pl-SP were up-regulated compared with Pl-P. Furthermore, most of the top10 up-regulated metabolites in Pl-SP presented strong antioxidant or against melanin synthesis activity. Hence, we further investigated the antioxidant capacity and tyrosinase inhibition assay to verify biological activity differences among three parts of the flowers.

The results of DPPH radical scavenging activities, ferric reducing antioxidant power (FRAP), tyrosinase monophenolase and diphenolase inhibition activity were displayed in Table 2. All the experiments were independently repeated three times (n = 3). The FRAP in Pl-SP was significantly higher (p < 0.05) than that in Pl-P and Pl-S. Additionally, IC_50_ values of tyrosinase monophenolase inhibition activity in Pl-SP were significantly lower (*p* < 0.05) than that in Pl-P and Pl-S. Similarly, the IC_50_ values of DPPH radical scavenging and tyrosinase diphenolase inhibition activity in Pl-SP were numerically lower than those in Pl-P and Pl-S.

**Table 2:** Biological activity of petal, stamen petaloid tissue and stamen of *P*. *lactiflora*.

Sample	Anti-oxidant capacity		Tyrosinase inhibition activity	
	FRAP (μmol/g)	DPPH (IC_50_, mg/mL)	Monophenolase (IC_50_, mg/mL)	Diphenolase (IC_50_, mg/mL)
Pl-P	89.67*	0.26	6.16*	9.64
Pl-SP	132.14	0.15	2.27	5.02
Pl-S	86.53*	0.27	4.04*	8.64

* Indicate statistically significant differences compared with Pl-SP (*p* < 0.05).

## Discussion

*P*.*lactiflora* flowers are well known for the edible and medicinal benefits, and exhibits various beneficial health effects. Nevertheless, the application of *P*. *lactiflora* flowers always be restricted because its nutritional values and functional active ingredients have not been explored in depth. Until now, only a few kinds of metabolites of petals and stamens have been investigated. Flavonoids and phenolic acids [46] have been believed to be the main metabolites and the most effective constituents. In this study, we provide a comprehensive metabolic profile of 1102 compounds from *P*. *lactiflora* flowers. Based on the results, the main metabolites are flavonoids, phenolic acids, lipids and amino and derivatives. Monoterpenoides, such as paeoniflorin, oxypaeoniflorin, benzoylpaeoniflorin, lactiflorin, albiflorin and paeoniflorigenone, are the major characteristic compounds of *P*. *lactiflora* [47,48]. This is the first time that abundant compounds of triterpene, triterpene saponin and terpene are detected in *P*. *lactiflora* flowers. The results are consistent with previous studies and should have advanced our understanding of the chemical compositions of *P*. *lactiflora* flowers.

The formation of stamen petaloid tissue is a striking trend in the evolution of *P*. *lactiflora* floral morphology [30]. During the process of stamen transforming to petaloid tissue, flavonoids are the most crucial metabolites with largest number of DAMs and enriched KEGG pathways. It is probably related with the color change from yellow to pink in this process, that’s because differential expression of flavonoid biosynthetic genes and flavonoid accumulation can cause yellow formation in *P*. *lactiflora* flowers [49,50]. In addition, flavonoids are believed to be the main antioxidant components in *P*. *lactiflora* flowers [20]. Compared with stamen, five of the top10 up-regulated metabolites in stamen petaloid tissues are flavonoids. Compared with petal, there are 91 DAMs of flavonoids in stamen petaloid tissue (52 up-regulated and 39 down-regulated). Particularly, as the principal components of tea polyphenols, the relative contents of catechin and epicatechin in stamen petaloid tissue are significantly higher than those in petal and stamen. According to previous studies, tea polyphenols present excellent antioxidant and antibacterial activities [51,52], also can be used to prevent and treat diseases such as skin photoaging [53], cancer [54,55] and obesity [56].

Phenolic acids [23,57], tannins [58,59] and alkaloids [60] are also dominant antioxidant substances in *P*. *lactiflora* flowers. Totally, 43 phenolic acids, 16 alkaloids and 7 tannins are detected with significantly high levels in stamen petaloid tissue compared with petal. Some of the highly accumulated compounds are shown to possess beneficial bioactivities, including four phenolic acids and three alkaloids in the top10 up-regulated metabolites. For example, N-p-coumaroyl-N’-feruloylputrescine [61], an alkaloid, shows antioxidant and anti-melanin production activities. Besides, both arbutin [62] and its derivatives 6’-p-coumarylarbutin [63] have strong inhibitory effect on human tyrosinase activity and arbutin is commonly used as a powerful skin whitening agent in cosmeceuticals [64]. Also, 2’,6’-dihydroxyacetophenone [65] is a bioactive phenolic acid with anticancer properties. In addition, camelliaside A can relieve burns via inhibiting inflammation and enhancing collagen synthesis [66], and shows obvious neuroprotective activity [67].

What’s more, in vitro test indicated that the antioxidant capacity and tyrosinase inhibition activity of stamen petaloid tissue are stronger than those of petal and stamen. These results largely depend on those higher content compounds in stamen petaloid tissue, especially catechins, arbutin and paeoniflorin that play a prominent part in antioxidant and tyrosinase inhibition activity [16]. To some extent, the formation of stamen petaloid tissue have determined the enrichment of crucial bioactive components and the higher pharmaceutical activities, revealing that the stamen petaloid tissue may become a preferable pharmacologically active resource. Considering the wide varieties of double-petals *P*. *lactiflora*, even though these findings are of certain guiding significance, further studies on the metabolites and biological activity analysis in more varieties of double-petals flowers are needed.

## Conclusion

In this study, a UHLC-ESI-MS/MS-based metabolomics analysis was performed to study the metabolites differences among different parts of *P*. *lactiflora* flowers. A total of 1102 metabolites were identified, which greatly enriched the chemical components category in *P*.*lactiflora* flowers. The current results revealed that the biosynthesis of putative antioxidant metabolites, such as flavonoids, phenolic acids alkaloids and tannins were significantly enhanced in stamen petaloid tissue compared with that in petal. In addition, the color diversity of the appearance mainly occurred in the phenomenon of stamen petaloid, and this characteristic was associated with the flavonoid biosynthesis pathway. Surprisingly, stamen petaloid tissue presented stronger antioxidant activity and reducing melanin formation effects via verified experiments. Collectively, the present study contributes to a deeper knowledge of bioactive substance in *P*. *lactiflora* flowers. We highlight that stamen petaloid tissue may become a more valuable function food or skin care resource. In addition, our result also provides a reference for its further application in food, medicine, cosmetics and other fields.

## Supporting information

S1 Fig Heatmap of flavonoids in Pl-SP compare with Pl-P. (TIF)

S2 Fig Heatmap of phenolic acids in Pl-SP compare with Pl-P. (TIF)

S1 Table List and characteristics of the metabolites identified and quantified in *P*. *lactiflora* flower. (XLSX)

S2 Table List of differential accumulated metabolites in the Pl-P vs. Pl-SP dataset. (XLSX)

S3 Table List of differential accumulated metabolites in the Pl-S vs. Pl-SP dataset. (XLSX)

S4 Table Statistics of differentially accumulated metabolites among Pl-P (petal), Pl-SP (stamen petaloid tissue) and Pl-S (stamen). (XLSX)

S5 Table The results of DPPH radical scavenging activities. Include DPPH scavenging rate of different concentration, IC50 value and T-test result in different sheet. Orange coloured values indicate scavenging rate at different concentration, and yellow coloured values indicate IC50 values of different samples.
(XLSX)

S6 Table The results of ferric reducing antioxidant power (FRAP). Green coloured values indicate FRAP value of different samples.
(XLSX)

S7 Table The results of tyrosinase monophenolase inhibition activity. Include monophenolase inhibition ratio of different concentration, IC50 value and T-test result in different sheet. Orange coloured values indicate inhibition ratio at different concentration, and yellow coloured values indicate IC50 values of different samples.
(XLSX)

S8 Table The results of tyrosinase diphenolase inhibition activity. Include diphenolase inhibition ratio of different concentration, IC50 value and T-test result in different sheet. Orange coloured values indicate inhibition ratio at different concentration, and yellow coloured values indicate IC50 values of different samples.
(XLSX)
