# Supplementary figures and images for: Widely targeted metabolomics reveals stamen petaloid tissue of Paeonia lactiflora Pall. being a potential pharmacological resource
Source: PLoS One. 2022 Sep 2;17(9):e0274013. doi: 10.1371/journal.pone.0274013 (PMC9439255; doi:10.1371/journal.pone.0274013)

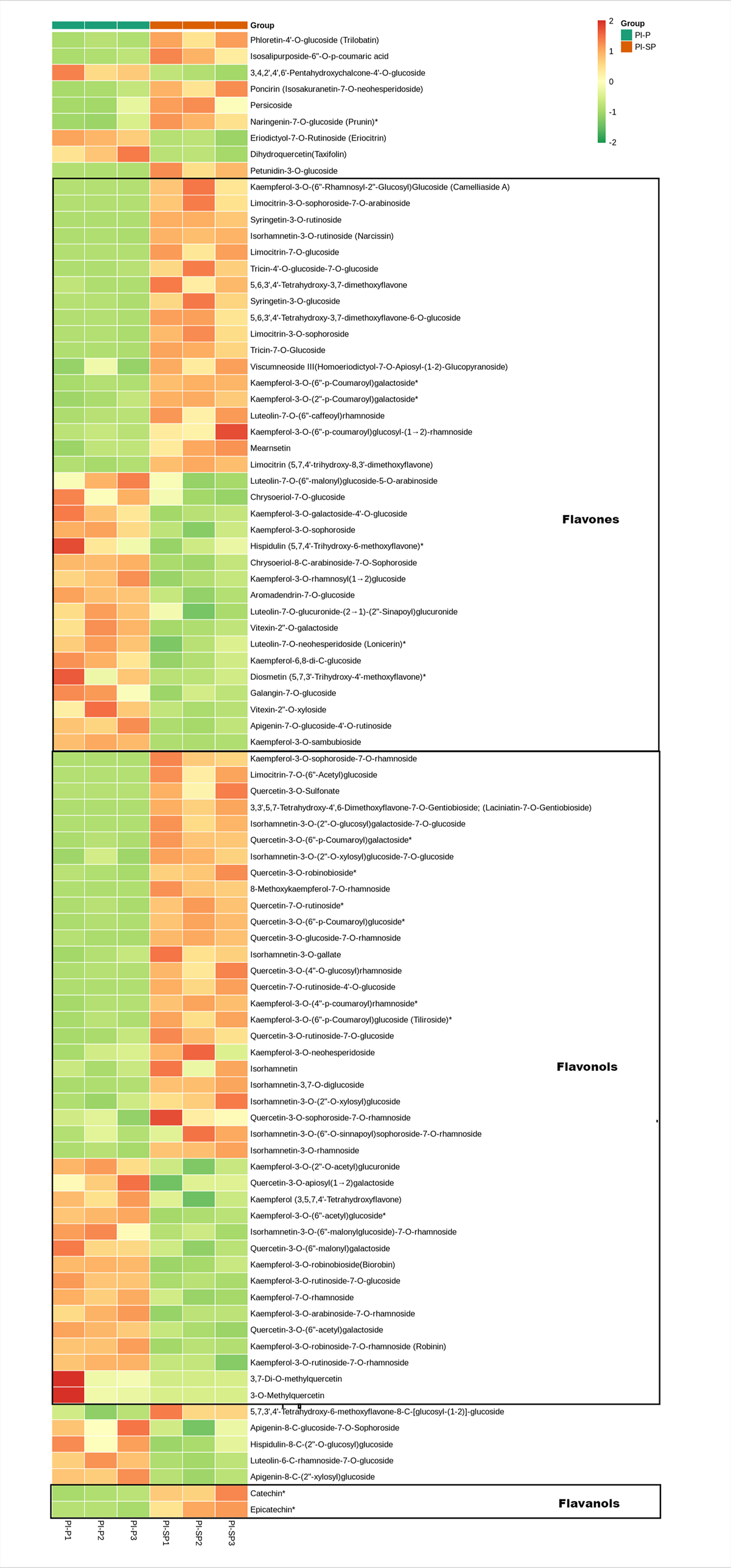

Supplement: S1 Fig — (TIF) [file pone.0274013.s001.tif]

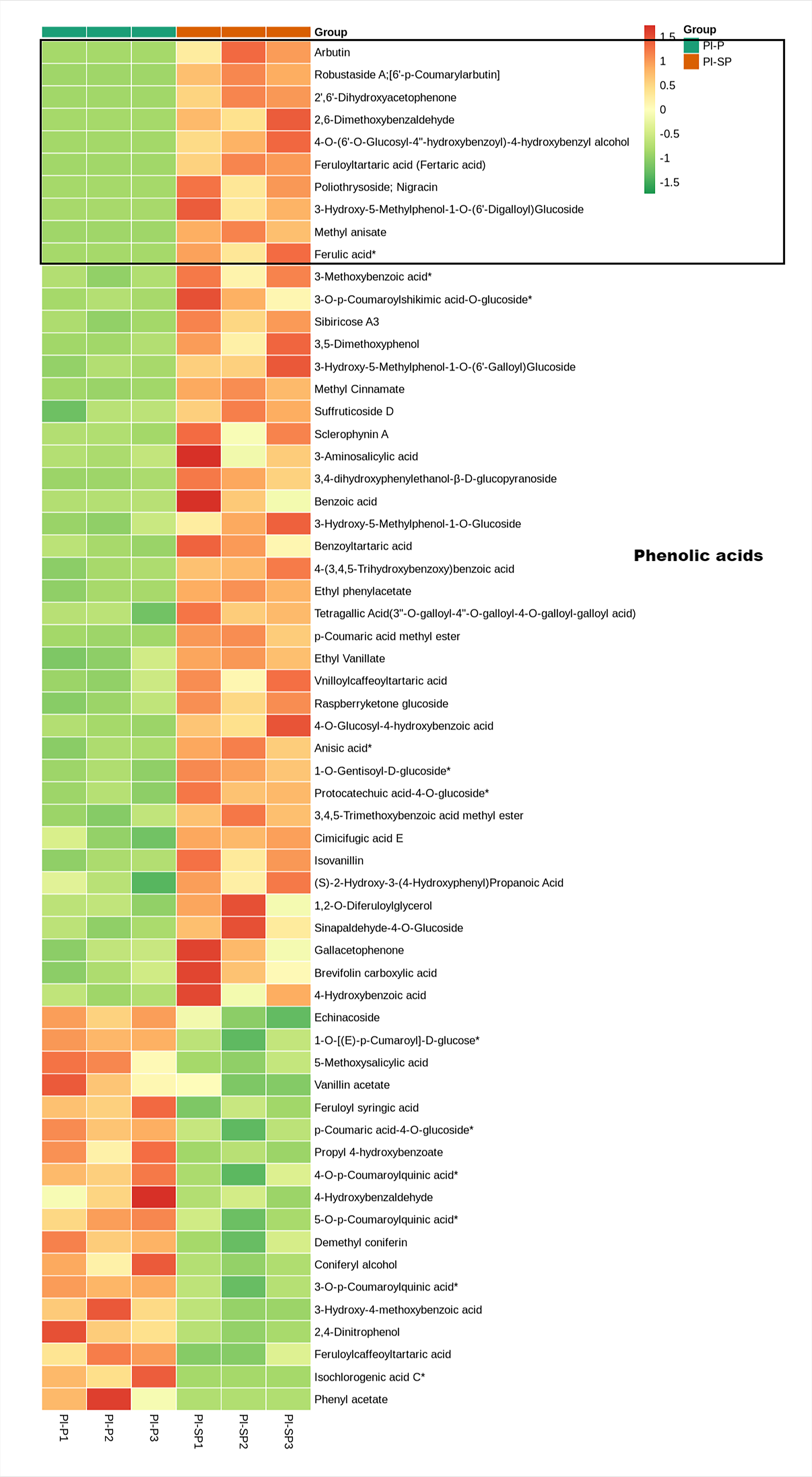

Supplement: S2 Fig — (TIF) [file pone.0274013.s002.tif]
